# Orthopedic surgery-induced cognitive dysfunction is mediated by CX3CL1/R1 signaling

**DOI:** 10.1186/s12974-021-02150-x

**Published:** 2021-04-15

**Authors:** Inja Cho, Jeong Min Kim, Eun Jung Kim, So Yeon Kim, Eun Hee Kam, Eunji Cheong, Minah Suh, Bon-Nyeo Koo

**Affiliations:** 1 Department of Anesthesiology and Pain Medicine, Yonsei University College of Medicine, 50-1, Yonsei-ro, Seodaemun-gu, Seoul, 03722 Republic of Korea; 2 Anesthesia and Pain Research Institute, Yonsei University College of Medicine, Seoul, Korea; 3 Department of Biotechnology, College of Life Science and Biotechnology, Yonsei University, Seoul, Republic of Korea; 4 Department of Biomedical Engineering, Sungkyunkwan University, 2066, Seobu-ro, Jangan-gu, Suwon-si, Gyeong gi-do 16419 Republic of Korea; 5 Center for Neuroscience Imaging Research (CNIR), Institute for Basic Science (IBS), Sungkyunkwan University, Suwon, Korea; 6 Biomedical Institute for Convergence at SKKU (BICS), Sungkyunkwan University, Suwon, 16419 South Korea; 7 Samsung Advanced Institute for Health Sciences & Technology (SAIHST), Sungkyunkwan University, Suwon, 16419 South Korea

**Keywords:** Postoperative pain, Postoperative cognitive dysfunction, CX3C chemokine receptor 1, Inflammation, GABA, Hippocampus

## Abstract

**Background:**

Postoperative pain is a common phenomenon after surgery and is closely associated with the development of postoperative cognitive dysfunction (POCD). Persistent pain and systemic inflammation caused by surgery have been suggested as key factors for the development of POCD. Fractalkine (CX3CL1) and its receptor, the CX3C chemokine receptor 1 (CX3CR1), are known to play a key role in pain and inflammation signaling pathways. Recent studies have shown that the regulation of CX3CR1/L1 signaling influences the development of various diseases including neuronal diseases. We determined whether CX3CR1/L1 signaling is a putative therapeutic target for POCD in a mouse model.

**Methods:**

Adult (9–11 weeks) male mice were treated with neutralizing antibody to block CX3CR1/L1 signaling both before and after surgery. Inflammatory and behavioral responses including pain were assessed postoperatively. Also, *CX3CR1* mRNA level was assessed. Hippocampal astrocyte activation, Mao B expression, and GABA expression were assessed at 2 days after surgery following neutralizing antibody administration.

**Results:**

The behavioral response indicated cognitive dysfunction and development of pain in the surgery group compared with the control group. Also, increased levels of pro-inflammatory cytokines and *CX3CR1* mRNA were observed in the surgery group. In addition, increased levels of GABA and increased Mao B expression were observed in reactive astrocytes in the surgery group; these responses were attenuated by neutralizing antibody administration.

**Conclusions:**

Increased CX3CR1 after surgery is both necessary and sufficient to induce cognitive dysfunction. CX3CR1 could be an important target for therapeutic strategies to prevent the development of POCD.

**Supplementary Information:**

The online version contains supplementary material available at 10.1186/s12974-021-02150-x.

## Background

Postoperative cognitive dysfunction (POCD) is a common neurological complication after surgery that is accompanied by impaired learning and memory [1, 2]. Transient cognitive impairment after surgery can lead to persistent cognitive dysfunction and dementia [3]. Although age, surgical duration, infection, and inflammation have been identified as the factors that lead to POCD, the exact physiological mechanism of POCD pathogenesis and optimal strategies for treatment or prevention of POCD remain unclear [4, 5]. Recently, neuroinflammation caused by systemic inflammation following surgery has been suggested as a key factor for the development of POCD [6, 7]. Persistent pain accompanied by surgery can induce systemic inflammatory mediators, and these factors lead to microglia activation. Consequently, microglia activation induces release of cytokines and other inflammatory mediators that have been associated with cognitive dysfunctions [8–11].

Cytokines and chemokines are important mediators in inflammation and the pathophysiology of inflammatory disease [12, 13]. Chemokines and other pain mediators regulate the interplay between glial cells and neurons in neuroinflammation and pain condition [14]. The neuronal chemokine fractalkine (CX3CL1) is a chemokine of the CX3C family and was first described as a potent attractant of immune cells [15, 16]. In the brain, CX3CL1 is expressed mainly in neurons while its receptor CX3CR1 is expressed mainly in microglia. CX3CL1/R1 interaction plays an important role in modulating glial activation in the central nervous system (CNS). CX3CL1/R1 interactions are also vital for many homeostatic processes, including the survival of blood monocytes [17], wound healing [18], and endothelial migration for immune surveillance [19, 20]. In addition, changes in CX3CL1/R1 immunoreactivity accompanied by microglia activation were detected after an ischemic event [21]. Although the association between CX3CL1/R1 signaling and POCD is established, the pathogenesis of postoperative cognitive impairment has not been elucidated. According to recent studies, the regulation of neurotrophic factors including cytokines and chemokines in the hippocampus, in response to systemic inflammation and pain after surgery, has been suggested as a key factor involved in cognitive impairment in POCD [22–24]. Consequently, alteration of neurotrophic factors in the brain enhances gamma-aminobutyric acid (GABA) release, which regulates neural activity by modulating inhibitory and excitatory responses [25, 26]. The hippocampus is a dynamic brain structure that consists of diverse neuronal cell types including neurons, astrocytes, and microglia, and is crucial for regulating cognitive function. Alterations of GABA levels in the hippocampus are involved in cognitive dysfunction in many CNS diseases [27, 28]; hence, GABA is also a target for the treatment of cognitive impairment in schizophrenia and epilepsy [29, 30].

Tibial fracture (TF) surgery is one of the most widely used animal models for POCD [22, 24]. In this study, we examined changes in CX3CR1 expression levels in the TF-induced POCD model and the potential therapeutic effect of CX3CL1 as a new treatment candidate for alleviating cognitive impairment by regulating inflammation. Also, this is the first study to show that the regulation of CX3CR1/L1 can improve postoperative cognitive impairment via modulation of GABA expression, which is mediated by the regulation of astrocyte activation.

## Materials and methods

### Animals

All procedures involving animals were approved by the Institutional Committee for the Care and Use of Laboratory Animals at Yonsei University Health System. Adult (9–11 weeks) male C57BL/6 (Orient, Seongnam, GyeongGi-Do, South Korea) mice were used for these experiments. All mice were housed in a controlled animal facility at Yonsei University. The mice were housed in groups of five per cage on a 12-hour light/dark cycle with food and water available ad libitum.

### Study groups and surgery model

**Fig. 1:**
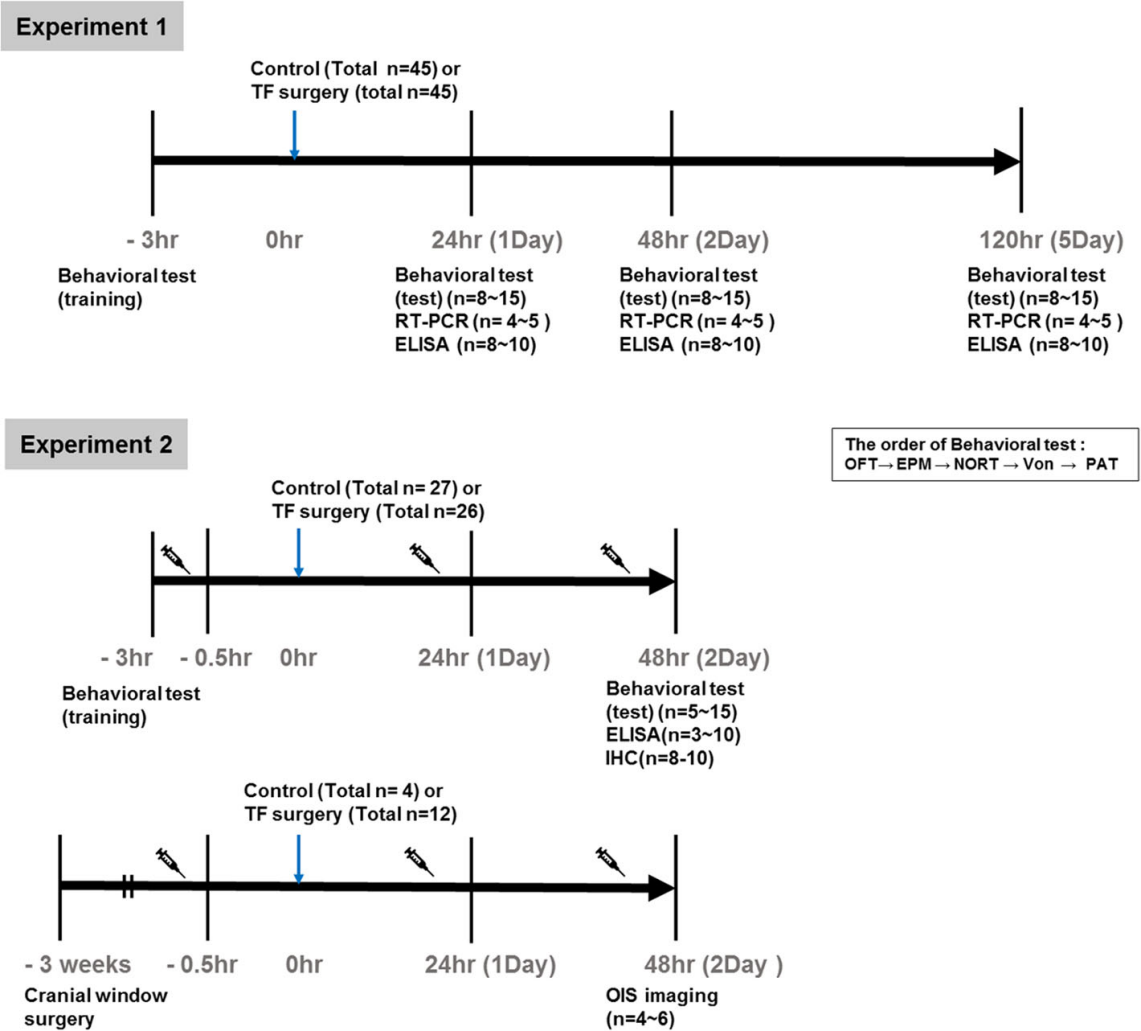
Schematic diagram of the experimental procedure. In experiment 1, the mice underwent behavioral tests for the training phase 3 h before surgery and post behavioral tests 1, 2, and 5 days after surgery. Brain tissues were immediately harvested at the end of the behavioral testing, and ELISA analysis for TNF-α, IL-6, and IL-1β levels was performed. The level of *CX3CR1* mRNA was measured by RT-PCR. In experiment 2, neutralizing Ab was injected 30 min before TF surgery after behavioral training tests, and at the same time point 24 and 48 h after later. The mice performed behavioral tests for the training phase 3 h before surgery and post behavioral tests 2 days after surgery. The brain tissues were harvested and ELISA and immunohistochemistry was performed after the behavioral testing period. For OIS imaging, the mice received the cranial window implantation 3 weeks before surgery, and OIS imaging was performed 2 days after surgery

In experiment 1, the mice were randomly divided into three sets to investigate alteration of neurobehavioral function after surgery: control vs. 1 day after TF surgery, control vs. 2 days after TF surgery, and control vs. 5 days after TF surgery (*n* = 8~10). In experiment 2, the mice were randomly divided into four groups as follows: Control, TF surgery, neutralizing antibody (Ab)-TF surgery and, neutralizing Ab-control (without surgery) (total *n* = 5~13 per group). A schematic diagram of the experimental procedure is shown in Fig. 1. In the TF surgery group, TF surgery was performed as previously described with small modifications [8, 31]. Briefly, mice received a fracture under isoflurane anesthesia (2.0–3.0 % inspired concentration). An incision was made in the right tibia after disinfection, and an intramedullary fixation pin (*Ø* = 0.38 mm) was inserted into the bone marrow cavity at the level of the tibial tuberosity to create an osteotomy. Mice in the neutralizing Ab-TF surgery group received neutralizing Ab (CX3CL1, 4 μg/mouse, dissolved in 96 μL saline, i.p.; R&D Systems, Minneapolis, MN, USA) 30 min before TF surgery after behavioral training tests, and same time point 24 and 48 h later [32]. Mice in the TF surgery group were injected with the same volume (100 μL) of saline. Control group was anaesthetized with isoflurane without any surgical procedure for approximately 10–15 min.

### Neurobehavioral tests

As depicted in the schematic diagram (Fig. 1), the mice performed the training phase of the behavioral test 3 h before surgery and the post behavioral tests 1, 2, and 5 days after surgery. All behavioral tests were conducted in the same behavioral testing room at the same time. To eliminate the influence of environmental novelty of testing room conditions, the mice were placed in their home cage in the behavioral testing room for a minimum of 30 min in the absence of the experimenter. The mice underwent multiple behavioral tests in the following order: open field test, elevated plus maze test, novel object recognition test, von Frey test, and finally, passive avoidance test. Mice were tested in a random order. During an interval time of 5 min between each test, the behavioral apparatus was cleaned with distilled water and 70% ethanol to eliminate olfactory cues.

An open field test was performed to assess exploratory activity and the level of anxiety. Mice were tested in a square open-field arena (40 × 40 × 40 cm) and behavior was recorded for 5 min. The total distance traveled and the time spent in the center zone were assessed.

For assessing the memory function of mice, the elevated plus maze test was performed. The maze was 50 cm in height and consisted of two open arms (31 × 6 × 1 cm) and two enclosed arms (31 × 6 × 15 cm), with a central open square area (5 × 5 × 1 cm). While the mice were allowed 5 min to explore the setting, we recorded the time to enter the closed arms. Based on the natural aversion of mice to high and open spaces, the learning index is calculated as the difference in entering time (in which the mouse first moves from the open arms to the closed arm) of training period − entering time (in which the mouse first moves from the open arms to closed arm) of test period = learning index [8, 33–36].

To evaluate the recognition memory ability of mice, the novel object recognition test was performed in a square arena (40 × 40 × 40 cm) for 5 min. In the familiarization phase, the mice were allowed to explore two equal objects (●+●) for 5 min. In the test phase, one object was changed to a novel object (●+■) placed within the same physical location, and the mice were allowed to explore both objects for 5 min. During both the familiarization and test phases, the time spent exploring each object was measured and recorded. Recognition memory ability was expressed as a percentage of novel object discrimination during total exploration time.

The passive avoidance test was used to evaluate learning and memory. We used a rectangular chamber divided into dark and lit compartments. During the acquisition phase, mice were placed into the lit compartment and allowed to explore freely for a maximum of 5 min. When mice entered the dark compartment, they received an electric shock (0.5 mA) for 3 s. In the test phase, mice were placed in the lit compartment and the time taken to enter the dark compartment was recorded.

All neurobehavioral tests were recorded on video and analyzed with an image analyzing system (SMART v2.5.21 software and SMART video Tracking system, Panlab Harvard Apparatus, Barcelona, Spain).

To evaluate mechanical allodynia, the von Frey filament test (Bioseb Inc., Vitrolles, France) was performed in accordance with a previously described method [37]. Briefly, the mice were individually placed in an acrylic box with a metal mesh grid. Different von Frey filaments, ranging from 2 to 60 g, were gradually applied for up to 3 s to the mid-plantar area of the right hindpaw in an ascending manner. Rapid retraction, shaking, or licking of the hind paw at least three times in five tests was considered to indicate a positive reaction.

### Enzyme-linked immunosorbent assay (ELISA)

Animals were fully anaesthetized and perfused with saline. The hippocampus, the prefrontal cortex, and amygdala regions were isolated. To measure IL-1β, IL-6, and TNF-α levels, tissues were lysed using tissue protein extraction reagent (T-PER® Tissue Protein Extraction Reagent, ThermoFisher Scientific™, Waltham, MA, USA) containing a protease and phosphatase inhibitor cocktail (100X Halt protease and phosphatase inhibitor cocktail, #1861281 Thermo Scientific™) at 6,000×*g* for 10 min at 4 °C. Samples were analyzed with a high-sensitivity mouse ELISA kit (Quantikine® ELISA, R&D Systems) according to the manufacturer’s instructions. After the reaction was complete, the results were read at 450 nm using a microplate reader.

### RT-PCR

The brains from mice in each group were rapidly dissected, collected, and stored at – 70 °C until real-time PCR was performed. Total RNA was extracted using the HiGene™ Total RNA Prep kit (BIOFACT, Daejeon, Korea) following the manufacturer’s instructions. Tissues (< 30 mg) were lysed with β-mercaptoethanol and proteinase K. After the samples were centrifuged at 18,000×*g* for 3 min at 4 °C, ethanol (100 %) was added and the mixture was vortexed for 30 s. The RNA was eluted with 50–100 μL RNase-free water. The purity of RNA was measured at 260/280 nm using NanoDrop® ND-1000 (Thermo Fisher Scientific). The primers were purchased from Bioneer (Daejeon, Korea) (CX3CR1 Forward, 5′ - CAGCATCGACCGGTACCTT - 3′; Reverse, 5′ - GCTGCACTGTCCGGTTGTT - 3′). PCR was performed in a total reaction mixture volume of 20 μL, which was composed of One-step SYBR RT-PCR Buffer, PrimeScript 1 step Enzyme Mix 2, ROX Reference Dye, forward primer and reverse primer, and sample RNA diluted in RNase-free distilled water according to manufacturer’s protocol. One-step real-time PCR was performed using the One-step SYBR PrimeScript RT-PCR Kit II (Perfect Real Time; Takara Bio Inc., JAPAN) on an ABI StepOnePlus Real-Time PCR system (Applied Biosystems, Thermo Fisher Scientific). The PCR cycling system was set as follows: reverse transcription was performed for 5 min at 42 °C and for 10 s at 95 °C. The PCR reaction was performed for 40 cycles of 5 s at 95 °C and for 34 s at 60 °C. The final step was performed at 95 °C for 15 s, at 60 °C for 1 min, and at 95 °C for 15 s. Cycling threshold values were normalized to the cycling threshold values of β-actin. The results were analyzed with the StepOneSoftware v2.3 (Thermo Fisher Scientific).

### Optical imaging system

To evaluate cerebral blood volume (CBV), hemodynamic responses to whisker stimulation were recorded with an optical imaging system (Imager 3001-Celox, Optical Imaging). The cortex was exposed for chronic imaging via cranial window surgery [38]. The animal was allowed to recover for 3 weeks to avoid inflammation due to the cranial window implantation procedure. The cranial window was illuminated with an LED lamp (CLS150, Leica Microsystems). Images were taken with a 10-Hz frame rate using a CCD camera (Photonfocus AG) through 50 mm tandem lenses at 546 nm wavelength, an isosbestic wavelength in the absorption spectra of oxyhemoglobin and deoxyhemoglobin, to assess CBV changes. The reflected light was filtered with a 546 ± 30 nm bandpass filter to measure the total amount of hemoglobin (Hbt). For whisker forelimb stimulation, air-puff stimulation was applied for 15 s. The activated area within the whisker cortex was identified and a region of interest (ROI) was placed to assess CBV changes over the course of 60 s. Optical intensity changes in these ROIs were computed for each trial with the baseline defined as the 5-s period preceding the whisker stimulus onset. Pixel changes compared to the baseline were calculated and plotted over the 60 s period.

### Immunofluorescence staining

Animals were fully anaesthetized and perfused with saline and 4% formaldehyde. The brain was post-fixed with 4% formaldehyde in PBS for one day at 4 °C. Afterward, the brain was dehydrated with 30% sucrose in PBS for at least 3 days and then it was rapidly frozen with OCT (optimal cutting temperature) compound. Coronal sections (20 μm) were cut using a cryotome. The sectioned tissues were incubated with 3 % bovine serum albumin and 0.3% Tx-100 in PBS for 1 h at room temperature and incubated with the primary antibody (GFAP, monoamine oxidase B (Mao B) and Iba-1; Cell Signaling Technology) overnight at 4 °C. The fluorescence-conjugated secondary antibody (1:200; Jackson) was applied for 1 hour at room temperature. Tissues were mounted with Vectashield with 4′,6-diamidino-2-phenylindole (DAPI; Vector Laboratories, USA). The stained sections were observed using laser scanning (LSM 700 confocal) microscopy. For semi-quantitative estimation of the staining intensity of Mao B expression, the fluorescence intensity of Mao B-stained regions was analyzed by calculating their mean fluorescence intensity using Zen 2010 software (Carl Zeiss). Sholl analysis was performed using Image J (FIJI-Image J).

### Statistical analysis

All quantitative data were analyzed using Prism software v7.0 (GraphPad Software, USA). Statistically significant differences between two groups were determined using Welch’s *t*-tests. Data from multiple groups were analyzed using one-, two-way ANOVA (Supplementary Figure S2-4) followed by Tukey post hoc tests for multiple comparison. Results are expressed as group mean ± SME. Statistical significance was set at *p* < 0.05.

## Results

### Cognitive dysfunctions and inflammatory responses in TF-induced POCD mice

**Fig. 2:**
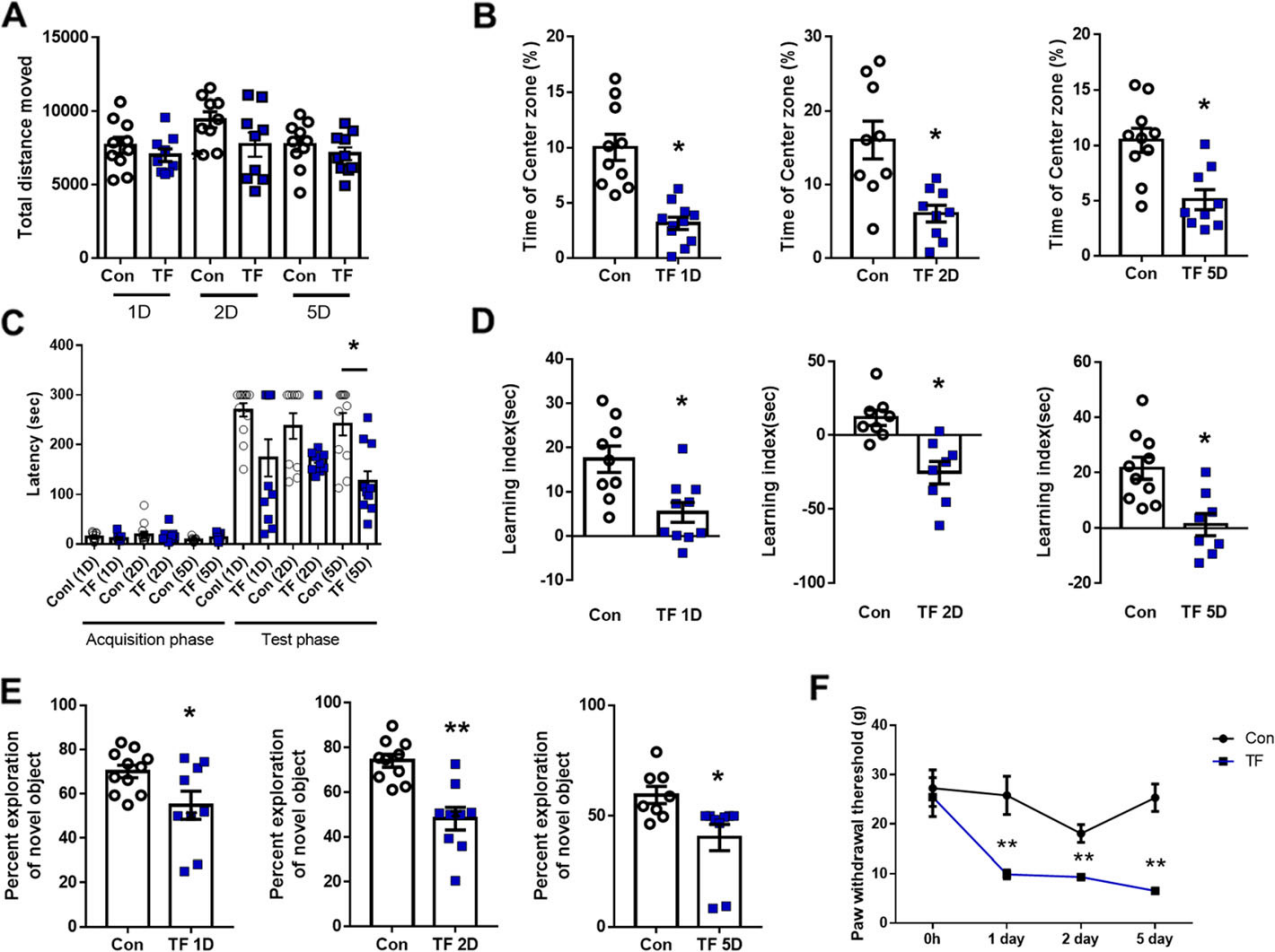
Assessment of neurobehavioral abilities of POCD mice. **a** Total distance traveled and **b** percentage of time in center zone in the open-field test. **c** Latency to enter the dark compartment in the passive avoidance test. **d** Learning index (entering time in which a mouse first moves from the open arms to closed arm during the training period − entering time in which a mouse first moves from the open arms to closed arm during the test period) in the elevated plus maze test. **e** Discrimination time rate for novel objects in the test phase of the novel objective recognition test (unpaired *t*-test, *n* = 8~11 per group). **f** Mechanical allodynia was performed using the von Frey test (two-way ANOVA with Tukey’s post hoc test, *n* = 15 per group). Con, normal control group; TF, tibial fracture surgery group; 1D, 1 day after surgery; 2D, 2 days after surgery; 5D, 5 days after surgery; AP, acquisition phase; TP, test phase; *p < 0.05 compared to the control; **p < 0.01 compared to the control

To assess the learning and memory ability in TF-induced POCD mice, we performed longitudinal neurobehavioral tests following surgery. In the open-field test, the total distance traveled did not show a significant difference at 1, 2, and 5 days after surgery compared to the control group (Fig. 2a; *t*-test, *p* = 0.3446, *p* = 0.1095, *p* = 0.3508, respectively); however, the percentage of time in the center zone was significantly decreased post-surgically (Fig. 2b; *t*-test, *p* = 0.0002, *p* = 0.0046, *p* = 0.0015, respectively, *n* = 9–10). In the passive avoidance test, the latency time was significantly decreased at 1, 2, and 5 days after surgery during the test phase (Fig. 2c; *t*-test, *p* = 0.0312, *p* = 0.0326, *p* = 0.0011, respectively). In addition, the learning index in the elevated plus maze test was significantly decreased 1, 2, and 5 days after surgery compared to the control group (Fig. 2d; *t*-test, *p* = 0.0058, *p* = 0.0015, *p* = 0.0025, respectively). The exploring rate for new objects in the novel objective recognition test was also significantly decreased at 1, 2, and 5 days after surgery compared to the control group (Fig. 2e; *t*-test, *p* = 0.0452, *p* = 0.0007, *p* = 0.0173, respectively). These data suggest that TF-induced POCD mice have cognitive impairments and increased anxiety. To evaluate pain after TF surgery, we performed the von Frey test. The mechanical paw withdrawal threshold (PWT) was decreased in the ipsilateral hindpaw from days 1 to 5 in TF-induced POCD mice, compared with that in the control mice (Fig. 2f; two-way ANOVA, *p* = 0.0038, *p* = 0.0024, *p* = 0.0002, respectively; *F*(3,112) = 12.26, *p* < 0.0001 [days], *F*(1,112) = 41.33, *p* < 0 .0001 [surgery], *F*(3,112) = 1.832, *p* = 0.1455 [interaction between days and surgery], *n* = 15), which indicated that pain lasted up to 5 days after surgery.

**Fig. 3:**
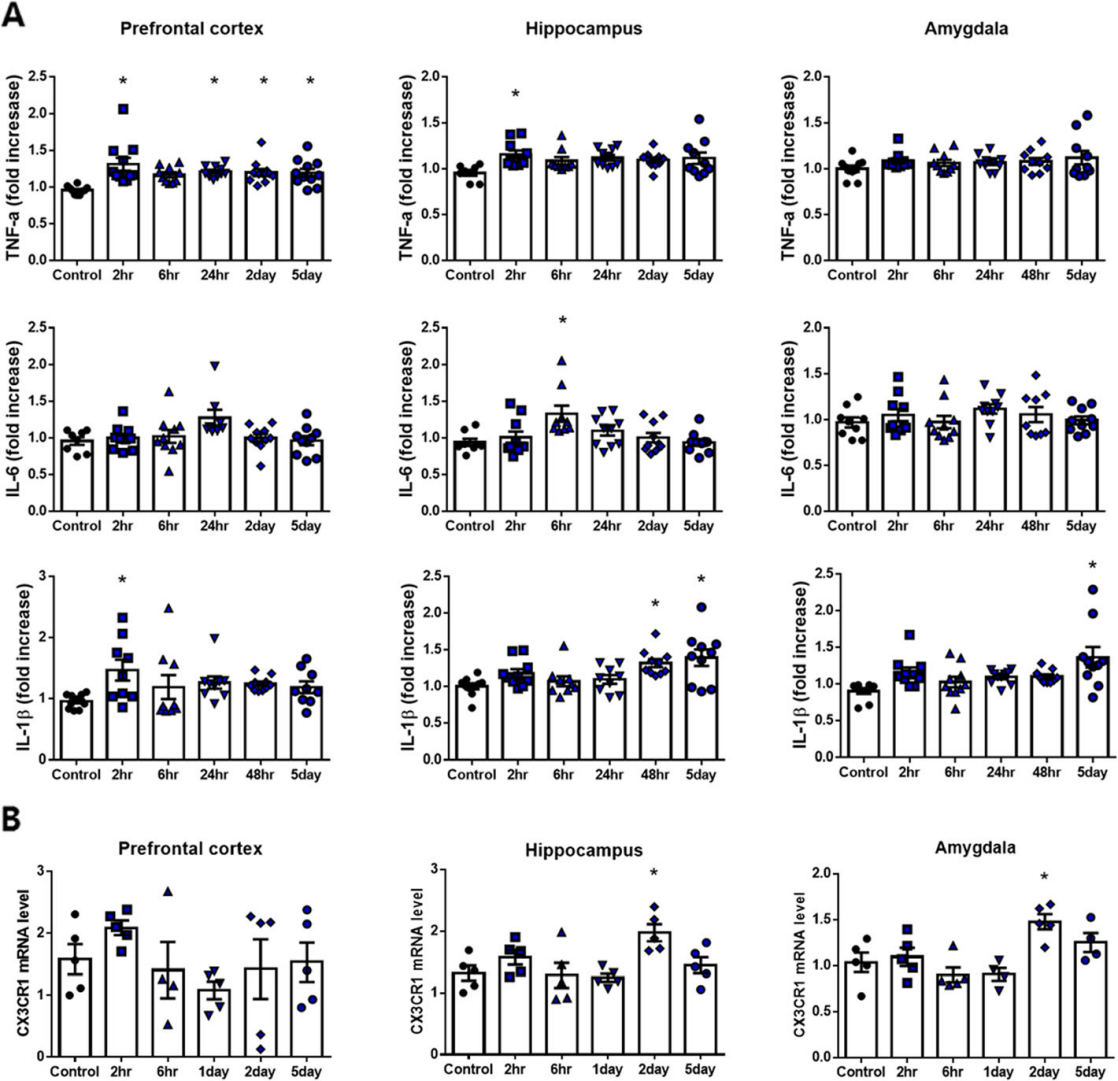
The level of pro-inflammatory cytokines and mRNA expression in POCD mice. **a** IL-1β, IL-6, and TNF-α in the prefrontal cortex, the hippocampus, and the amygdala at different time points after tibial fracture surgery (one-way ANOVA with Tukey’s post hoc, *n* = 8~11 per group). **b** The mRNA level of CX3CR1 in the prefrontal cortex, the hippocampus, and the amygdala at different time points after tibial fracture surgery (one-way ANOVA with Tukey’s post hoc, *n* = 4~5 per group). Control, normal control group; TF, tibial fracture surgery group; 2hr, 2 h after surgery; 6hr, 6 h after surgery; 24hr, 1 day after surgery; 2D, 2 days after surgery; 5D, 5 days after surgery; *p < 0.05 compared to the control

To evaluate the inflammatory response in the brain regions longitudinally following TF surgery, we measured the protein expression levels of TNF-α, IL-6, and IL-1β (Fig. 3a). Overall, IL-1β, IL-6, and TNF-α levels were increased in TF surgery groups. Especially during the early phase after surgery, such as at 2 h after surgery, the level of TNF-α was significantly higher in POCD mice compared to control (in hippocampus; one-way ANOVA, *p* = 0.0179, *n* = 10). Furthermore, the level of IL-1β was also significantly higher at 2 days after surgery compared to the control (in hippocampus; one-way-ANOVA, *p* = 0.0225, *n* = 10). Consequently, TF-induced POCD mice showed cognitive impairment and an elevated inflammatory response.

### Pain-mediated increase in CX3CR1 in POCD mice

We selected CX3CR1 as a chemokine related to pain and chemotaxis in the brain to analyze following TF surgery. Two days after surgery, *CX3CR1* transcription was significantly increased in the hippocampus (one-way ANOVA, *p* = 0.0247, *n* = 5) and amygdala (one-way ANOVA, *p* = 0.0213, *n* = 5) (Fig. 3b) based on RT-PCR analysis. Therefore, the subsequent experiments were performed 2 days after surgery in POCD mice.

### Attenuation of cognitive dysfunction and inflammatory response by blocking CX3CR1 signaling

**Fig. 4:**
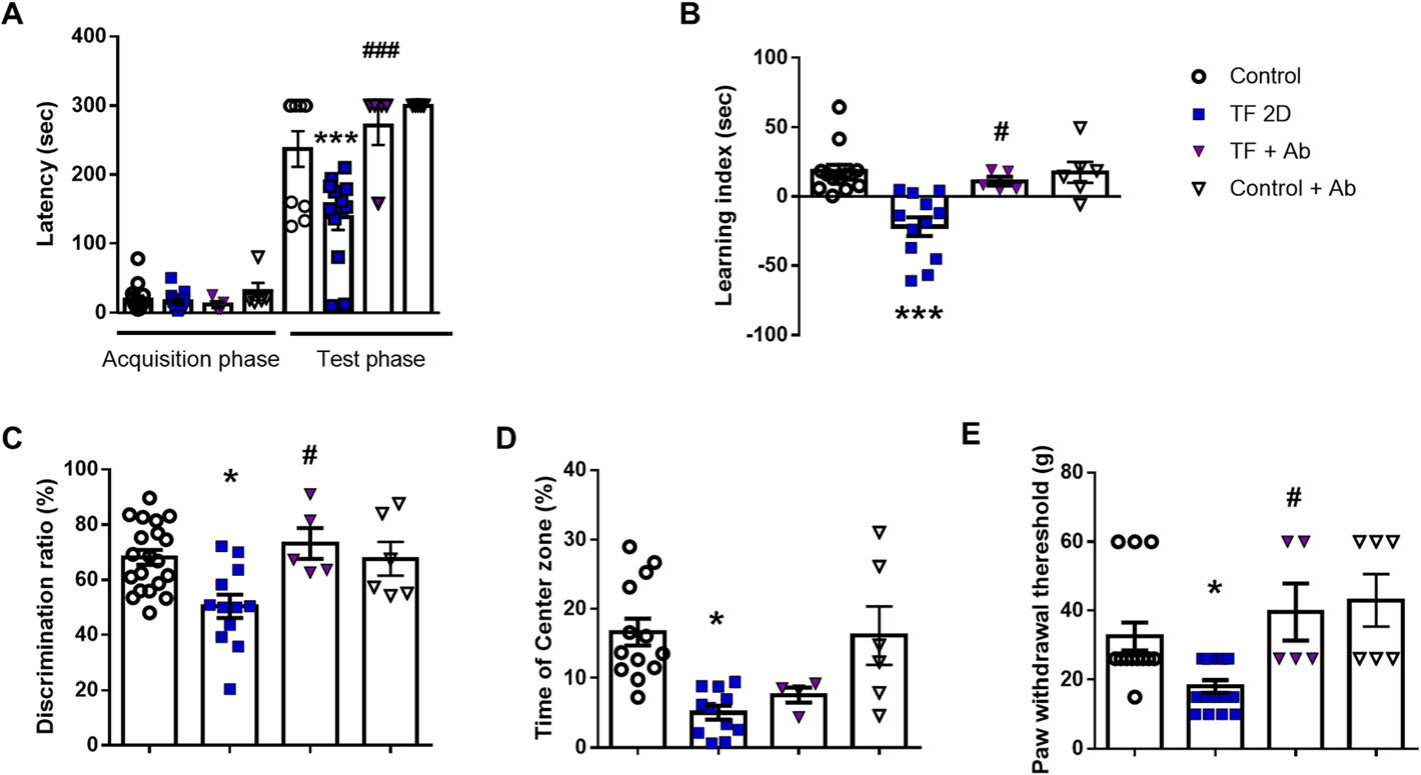
Evaluation of neurobehavioral abilities of POCD mice after inhibition of CX3CR1 signaling (one-way ANOVA with Tukey’s post hoc, *n* = 5~13 per group). **a** Latency to enter the dark compartment in the passive avoidance test 2 days after tibial fracture surgery. **b** Leaning index (transfer latency time to enter the closed arm of the training period − transfer latency time to enter the closed arm during the test period) in the elevated plus maze test. **c** Discrimination time rate for novel objects in the test phase of the novel objective recognition test. **d** Time rate in the center zone in the open-field test. **e** Mechanical allodynia was evaluated with the von Frey test. Control, normal control group; TF2D, 2 days after tibial fracture surgery group; TF+Ab, 2 days after tibial fracture surgery group injected with neutralizing Ab; Control+Ab, normal control group injected with neutralizing Ab; *p < 0.05 compared to the control; ^#^*p* < 0.05 compared to the TF2D; ^###^*p* < 0.0001 compared to TF2D

To evaluate the effect of inhibiting CX3CR1 signaling, we intraperitoneally injected the mice with neutralizing Ab (4 μg/mice) 30 min before surgery and 24 and 48 h after surgery. In the passive avoidance test, the latency was significantly increased in the Ab-injected group compared to the TF-induced POCD group (Fig. 4a, one-way ANOVA, *p* < 0.0001, *n* = 5). The learning index in the elevated plus maze test was also significantly increased in the Ab-injected group compared to the TF-induced POCD group (Fig. 4b, one-way ANOVA, *p* = 0.0130, *n* = 5). In addition, the exploration rate for new objects in the novel objective recognition test was significantly increased in the Ab-injected group (Fig. 4c, one-way ANOVA, *p* = 0.0135, *n* = 5). In the open-field test to assess anxiety levels, the rate of time spent in the center zone was significantly decreased in the TF-induced POCD group compared to the control group. However, it was non-significantly increased in the Ab-injected group (Fig. 4d, one-way ANOVA, *p* = 0.9066, *n* = 5). Mechanical PWT in the von Frey test was significantly increased in the Ab-injected group compared to the TF-induced POCD group (Fig. 4e, one-way ANOVA, *p* = 0.0222, *n* = 5).

**Fig. 5:**
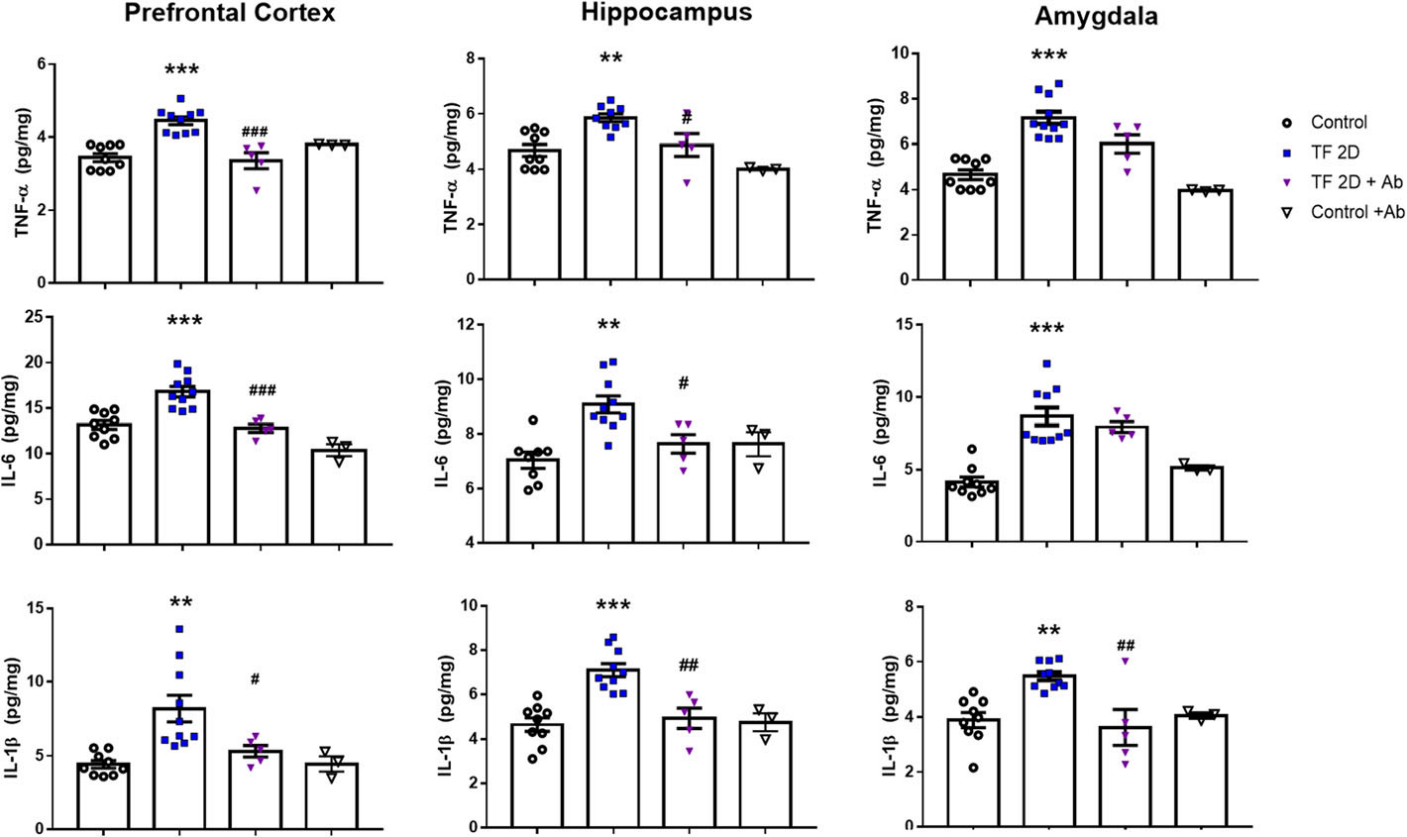
Anti-inflammatory effect of inhibition of CX3CR1 signaling in tibial fracture-induced POCD mice. The levels of the pro-inflammatory cytokines TNF-α, IL-6, and IL-1β in the prefrontal cortex, the hippocampus, and the amygdala (one-way ANOVA with Tukey’s post hoc, *n* = 3~10 per group). Control, normal control group; TF2D, 2 days after tibial fracture surgery group; TF+Ab, 2 days after tibial fracture surgery group injected with neutralizing Ab; Control+Ab, normal control group injected with neutralizing Ab. *p < 0.05; **p < 0.01; ***p < 0.0001 compared to the control; ^#^*p* < 0.05; ^##^*p* < 0.01; ^###^*p* < 0.0001 compared to TF2D

To confirm the effect of inhibition of CX3CR1 signaling on the inflammatory response in the brain regions, we measured the expression levels of TNF-α, IL-6, and IL-1β (Fig. 5). IL-1β expression was significantly decreased in the prefrontal cortex (*p* = 0.0447, *n* = 5), the hippocampus (*p* = 0.0016, *n* = 5), and the amygdala (*p* = 0.0026, *n* = 5) regions in the Ab-injected group compared to the TF-induced POCD group (one-way ANOVA, *n* = 10). Overall, decreased levels of inflammatory cytokines including TNF-α and IL-6 were detected in the brain regions of Ab-injected mice.

These data suggest that inhibition of CX3CR1 signaling attenuated cognitive dysfunction and inflammatory response in TF-induced POCD mice.

### Amelioration of the hemodynamic response to whisker stimulation following CX3CR1 signal blocking

**Fig. 6:**
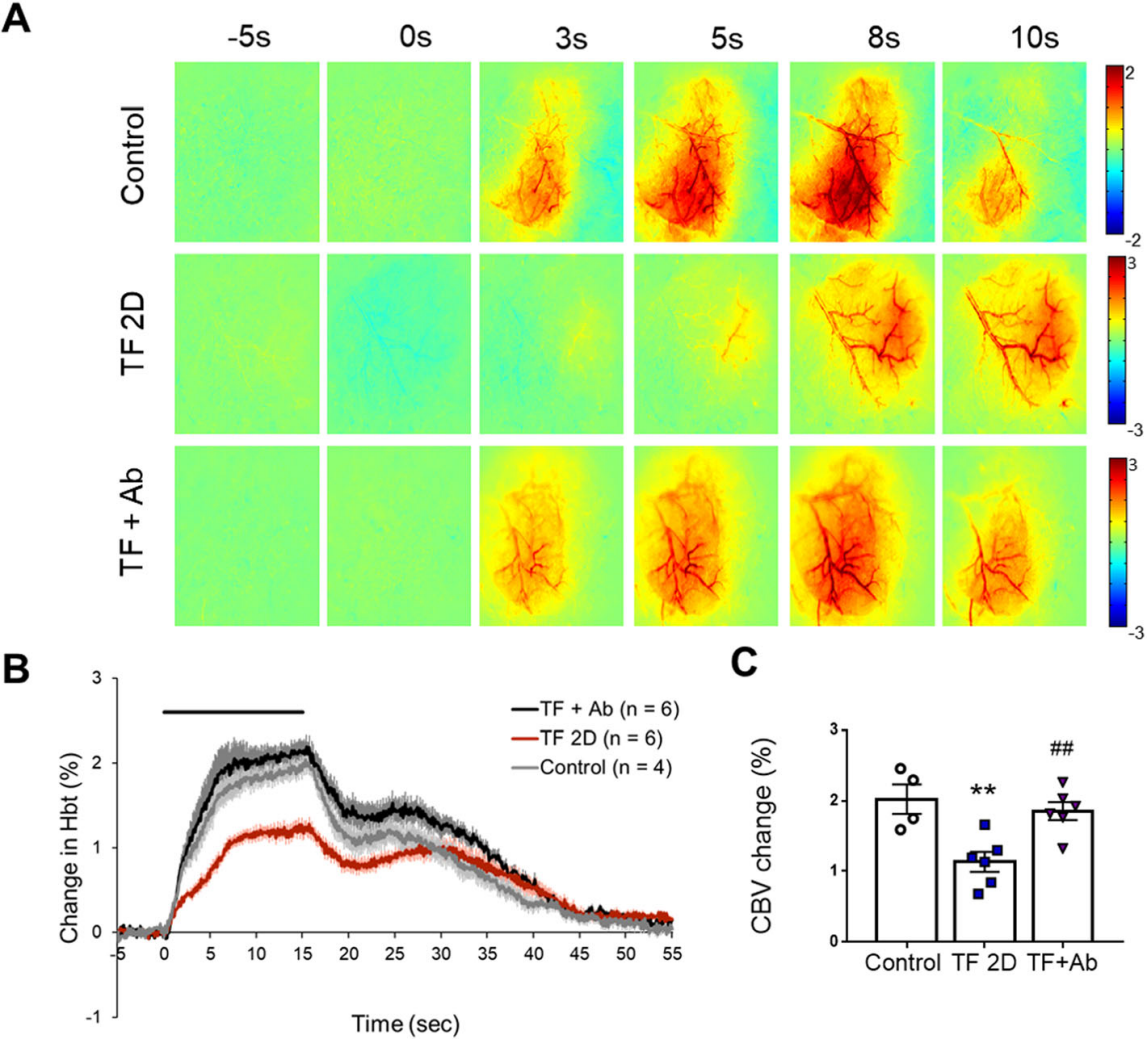
Impaired neurovascular coupling response to sensory stimulation in POCD. **a** Representative image of the intensity changes of the optical imaging signal during whisker stimulation. **b** Time course traces of relative changes in cerebral blood volume after stimulation. **c** Average of the maximum cerebral blood volume response (one-way ANOVA with Tukey’s post hoc test, *n* = 4 or 6 per group). Control, normal control group; TF2D, 2 days after tibial fracture surgery group; TF+Ab, 2 days after tibial fracture surgery group injected with neutralizing Ab. **p < 0.01 compared to the control; ^##^*p* < 0.01 compared to TF2D

The regulation of brain blood flow is critical for brain function [39, 40]. Abnormal changes in CBV responses can lead to neuronal dysfunction and accelerated neuronal damage and loss. Indeed, in some diseases accompanied by cognitive dysfunction, such as aging, dementia, and Alzheimer’s disease, changes in central blood flow and hemodynamics are found [41]. To assess hemodynamic responses to sensory stimulation in the POCD model, we performed optical intrinsic signal (OIS) imaging [42]. In the control group, robust and large changes of CBV were detected following whisker stimulation. The maximum CBV change (2.04 ± 0.41 %) was found at about 15 s (Fig. 6, *n* = 4). In POCD mice, the maximum CBV was found at a similar time point in the control group, but the peak CBV amplitude was significantly lower than in the control group (1.13 ± 0.38%, one-way ANOVA, *p* = 0.0048, *n* = 6). In the Ab-injected group, the peak CBV amplitude was significantly increased (1.87 ± 0.34%, one-way ANOVA, *p* = 0.0095, *n* = 6) compared to the TF surgery group.

### Normalization of astrocyte activation and GABA expression by blocking CX3CR1 signaling

**Fig. 7:**
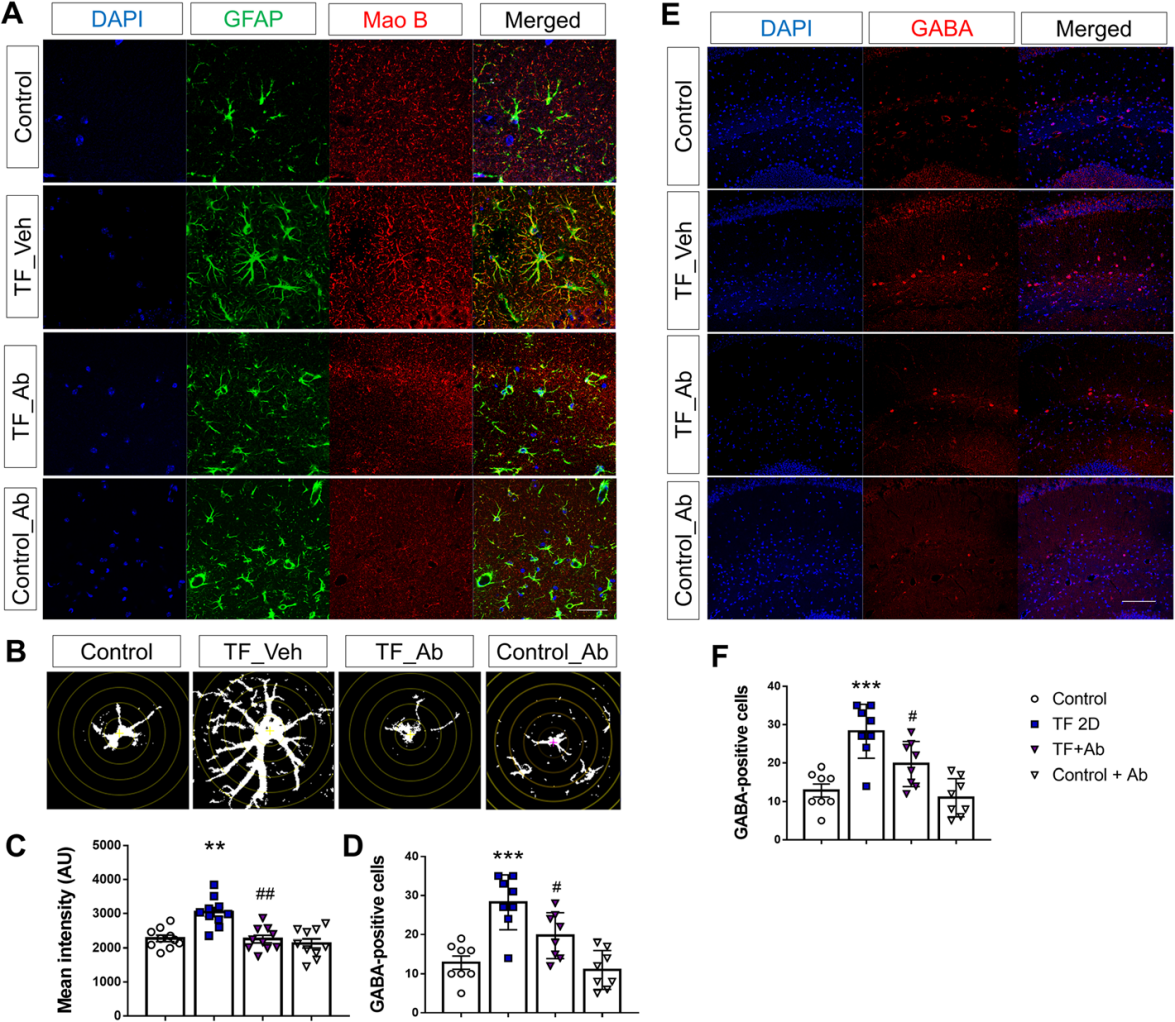
Effect of inhibition of CX3CR1 signaling on GFAP, Mao B expression, and GABA levels. **a** Representative immunofluorescence images for DAPI, GFAP, and Mao B. **b** Sholl analysis for a traced individual GFAP-positive astrocyte, which is superimposed over concentric circles (one-way ANOVA with Tukey’s post hoc test, *n* = 8 per group). **c** Intensity of Mao B images. **d** Quantification of the total number of intercepts with ramifications from Sholl analysis (one-way ANOVA with Tukey’s post hoc test, *n* = 10 per group). **a** Representative immunofluorescence images for DAPI and GABA. **b** Quantification of GABA-positive cells (one-way ANOVA with Tukey’s post hoc test, *n* = 8 per group). Control, normal control group; TF_Veh, 2 days after tibial fracture surgery group injected with saline; TF_Ab, 2 days after tibial fracture surgery group injected with neutralizing Ab; Control+Ab, normal control group injected with neutralizing Ab; scale bar, 20 μm, 100 μm. *p < 0.05; **p < 0.01; ***p < 0.0001 compared to the control; ^#^*p* < 0.05; ^##^*p* < 0.01; ^###^*p* < 0.0001 compared to TF2D

In the CNS, astrocytes play crucial roles including regulating synaptic homeostasis of neural networks. GABA is the main inhibitory neurotransmitter in the brain and plays a key role in regulating neuronal function [43]. Therefore, we also assessed alterations of astrocytes and GABA levels in the hippocampus. In TF-induced POCD mice, excessive reactive astrocytes were detected in the hippocampus (Fig. 7a) and Sholl analysis of individual GFAP-positive cells revealed significant differences compared to the control group (Fig. 7b, d; one-way ANOVA, *p* < 0.0001, *n* = 8 per group). Furthermore, levels of Mao B, a GABA synthesizing enzyme, were significantly increased in POCD mice (Fig. 7a, c; one-way ANOVA, *p* = 0.0003, *n* = 10 per group). In contrast, Ab injection resulted in the complete restoration of astrocyte morphology and Mao B expression to control levels (Fig. 7a, c; one-way ANOVA, *p* = 0.0002, *n* = 10 per group). We also assessed GABA levels and found a significant difference between control and POCD mice (Fig. 7e, f; one-way ANOVA, *p* < 0.0001, *n* = 8 per group). Increased GABA levels were detected in POCD mice compared to control mice while Ab injection normalized GABA levels (Fig. 7e, f; one-way ANOVA, *p* = 0.0292). However, mice treated with neutralizing Ab alone without surgery did not show a significant difference in GABA and Mao B expression as compared with control group. These results indicated that inhibition of CX3CR1 signaling by neutralizing Ab injection after TF surgery suppressed excessive astrocyte activation and aberrant GABA expression.

## Discussion

In this study, we revealed that transiently elevated CX3CR1 induces persistent pain and increased expression of pro-inflammatory cytokines after surgery, which consequently leads to astrocyte activation and increased GABA expression, eventually resulting in cognitive dysfunction. Moreover, we revealed the therapeutic effect of inhibiting CX3CR1 signaling in TF-induced POCD mice. In summary, inhibition of CX3CR1 signaling by injection of neutralizing Ab suppressed the increase in pro-inflammatory cytokines via inhibition of persistent pain that occurred after TF surgery; it also ameliorated cognitive impairment. Further, inhibition of CX3CR1 signaling restored basal CBV, astrocyte activation, and GABA levels.

The pathogenesis of POCD is still unclear, but one core pathophysiological mechanism of POCD involves neuroinflammation caused by postoperative systemic inflammation [44, 45]. The ineluctable systemic inflammation and pain after surgery induce microglia activation, which leads to induction of inflammatory mediators such as pro-inflammatory cytokines (TNF-α, Il-6, Il-1β) and chemokines (CX3CR1), which leads to persistent pain [8–11]. CX3CR1, as a receptor for CX3CL1, is a chemokine receptor known to be related to pain, and CX3CR1/L1 signaling is implicated in the pathogenesis of inflammatory diseases such as atherosclerosis, rheumatoid arthritis, and renal fibrosis by supporting monocyte recruitment and exacerbating tissue damage [46, 47]. Moreover, CX3CL1/R1 signaling regulates hippocampal neurogenesis, synaptic pruning, and synaptic plasticity in different pathological conditions [48]. In this study, we observed microglia activation (Supplementary Figure S1) and increased pro-inflammatory cytokines, including IL-6, IL-1β, and TNF-α in several brain regions. Further, 2 days after the surgery, increased CX3CR1 was detected compared to that in control. In the von Frey test, mechanical allodynia persisted for 5 days after the surgery. In addition, cognitive dysfunction was detected until 5 days after the surgery. These results indicated that increased CX3CR1 contributes to pain development, which in turn induces cognitive dysfunction.

Interestingly, we observed a change in astrocyte morphology, also known as activated astrocytes. Astrocytes play a crucial role in maintaining CNS homeostasis by cross-talk with neurons or microglia [49, 50]. However, in pathological conditions, the phenotype and function of astrocytes can change; these altered astrocytes are known as reactive astrocytes [51]. Indeed, abnormal astrocytes, such as in gliosis and excessive reactive astrocytes, cause synaptic imbalances that can induce cognitive impairment [52]. Recently studies have stated that astrocyte activation is associated with the development of pain by releasing signaling molecules [53, 54]. In addition, activated astrocytes in the brain regions related to emotion regulation, such as the primary somatosensory cortex, medial prefrontal cortex, and hippocampus, are associated with emotional dysfunction under chronic pain states [55–57]. Thus, based on these study results, our result is meaningful because an increased level of Mao B, a GABA synthesis associated enzyme, was observed in reactive astrocytes. Moreover, increased GABA expression was observed in the hippocampus Indeed, previous studies revealed aberrant astrocyte and GABA levels in an Alzheimer’s disease mouse model, which is characterized by cognitive impairment [58]. These results indicated that astrocyte activation was triggered by increased CX3CR1/L1 signaling, which consequently leads to increased GABA and proinflammatory cytokine expression in the hippocampus. Consequently, these changes may have affected cognitive dysfunction.

Previous studies show that high levels of CX3CR1/L1 signaling are directly proportional to increased levels of inflammatory factors [59]. Also, expression of CX3CR1 and CX3CL1 is regulated by inflammatory stimuli and is enhanced following exposure to lipopolysaccharides, pro-inflammatory cytokines, and during inflammatory conditions in endothelial and epithelial cells [21]. These studies may support the reason for the increased CX3CR1 or CX3CL1 through increased systemic inflammation after surgery. In contrast, deficient CX3CR1 signaling ameliorates the pathological condition in various models, such as those for spinal cord injury and hepatic encephalopathy [32, 60–62]. In neuropathic pain and mechanical hypersensitivity models, neutralizing CX3CL1 Ab attenuated peripheral hyperalgesia by decreasing microglia activation [63]. Similarly, we observed that inhibition of CX3CR1 signaling by CX3CL1 neutralizing Ab injection improved cognitive dysfunction and prevented the increase in pro-inflammatory cytokine levels as well as mechanical allodynia. Also, inhibited activation of microglia and astrocytes was observed, which normalized GABA expression. GABA is a major inhibitory neurotransmitter in the CNS and regulates the balance of excitation and inhibition required for cognitive ability. Aberrant GABA levels can impair working memory [28]. GABA has recently also been found to correlate with a marker of behavioral metrics, which increased the interest in measuring GABA levels to predict diseases [64, 65]. A previous study showed that glutamate decarboxylase (GAD), a GABA synthesizing enzyme, was regulated in a spinal cord injury model in CX3CR1-deficient rats [66]. In addition, another study reported that in an Alzheimer’s disease animal model, increased levels of GABA and Mao B in reactive astrocytes caused memory impairments [29]. Based on these data, we suggest that regulation of CX3CR1/L1 signaling by injection of neutralizing Ab after surgery affected the level of GABA expression and mechanical allodynia, which might consequently improve cognitive impairment.

Neurovascular coupling, i.e., the interactions between neurons, astrocytes, and cerebral blood vessels [67, 68], affects normal brain function and the release of neurotransmitters, neuromodulators, and vasoactive mediators [69]. The measurement of hemodynamic responses by utilizing OIS and functional MRI provide a means to assess neurovascular coupling. Hemodynamic responses, including CBV, are correlated with various factors that regulate neuronal activity and energy production. GABAergic inhibitory neurons are known to play an important role in regulating hemodynamic responses. In the cortex, exogenous GABA has been shown to regulate CBV [70] but not in the cerebellum. Previous studies have revealed impaired CBV in dementia and Alzheimer’s disease. In line with these reports, our results revealed that decreased CBV in the POCD model indicate decreased hemodynamic responses and neurovascular coupling [40]. Clinical data also showed that the level of occipital GABA inversely correlates with CBV [71]. Collectively, we suggest that cognitive dysfunction following TF surgery is closely related to CBV reduction and alterations in neuronal activation, i.e., excitatory/inhibitory imbalance. Furthermore, our results revealed that CBV amplitude recovered in the Ab-injected surgery group. Thus, TF-induced POCD mice displayed decreased hemodynamic responses to sensory stimulation, which is likely to be related to brain dysfunctions such as cognitive impairment, whereas the inhibition of CX3CR1/L1 signaling attenuated the reduction in hemodynamic responses following sensory stimulation. Consequently, our results suggest that the CX3CR1/L1 signaling pathway-mediated GABA imbalance and increased inflammatory cytokines following TF surgery could be the main causes of cognitive dysfunction.

There are many potential diseases in which inhibiting the CX3CL1/R1 signaling pathway may be promising. For instance, after peripheral nerve injury [72, 73], CX3CR1 is upregulated in spinal microglia, and a spinal nerve transection model shows increased CX3CL1 expression in astrocytes [72]. In addition, intrathecal administration of CX3CL1 or CX3CR1 neutralizing Ab has protective effects in attenuating neuropathic pain behaviors in peripheral injury models by reducing p38 MAPK phosphorylation [74, 75]. In a bone cancer model, the development of pain occurs concurrently with microgliosis and an increase in the expression of microglial CX3CR1 and p-p38. Via intrathecal administration of a CX3CR1 neutralizing Ab, the onset of pain is significantly attenuated [76, 77]. Furthermore, pain-mediated neuronal signaling and neuroinflammation were ameliorated by CX3CL1 neutralizing Ab administration in multiple sclerosis and hepatic encephalopathy models [60, 78]. Not only have neutralizing Ab and modified CX3CL1 been used in proof-of-concept preclinical studies, CX3CR1 antagonists have also shown anti-inflammatory activities in both mice and humans [79, 80]. Likewise, several studies have reported positive effects on pathological mechanisms by inhibiting CX3CR1/L1 signaling via neutralizing Ab, a mechanism that is related to inhibition of microglia activation and inflammation response.

### Limitations

Several studies have reported the protective effect of CX3CR1/L1; however, this is the first study to state that the regulation of CX3CR1/L1 can improve cognitive impairment after surgery via modulation of GABA expression, which is mediated by the regulation of astrocyte activation. However, there is a limitation to our study. As an additional control for the Ab-injected group, we should have had an IgG-injected group. Instead, we confirmed that treatment with neutralizing Ab alone without surgery did not show a significant difference as compared with control group.

Although further studies are needed to investigate the detailed molecular mechanism underlying astrocyte activation and GABA signaling induced by changes in the CX3CR1/L1 interaction during surgical pain, this study is the first to show that microglia activation by increased postoperative systemic inflammation induces a change in CX3CR1/L1 signaling and causes persistent pain and astrocyte activation, which consequently alters the level of neurotransmitters such as GABA, eventually leading to cognitive impairment. This study further confirmed the potential therapeutic effects of regulating CX3CR1/L1 signaling to prevent cognitive impairment in POCD, via modulation of persistent pain and inflammatory response, as well as GABA signaling, by regulating astrocyte activation.

## Conclusion

Transient increased CX3CR1 after surgery is both necessary and sufficient to induce cognitive dysfunction. Neutralizing antibody blockade of CX3CR1/L1 signaling prevented POCD by reducing pro-inflammatory cytokine expression as well as regulating GABA levels in the hippocampus by regulating astrocyte activation. Inhibition of CX3CR1/L1 signaling could be an important target for therapeutic strategies to prevent the development of POCD.

## Supplementary Information

**Additional file 1: Figure S1.** The expression of Iba-1 in hippocampal tissue.

**Additional file 2: Figure S2.** Data in Figure 4, analyzed by two-way ANOVA followed by Tukey’s post hoc test. **Figure S3.** Data in Figure 5, analyzed by two-way ANOVA followed by Tukey’s post hoc test. **Figure S4.** Data in Figure 7, analyzed by two-way ANOVA followed by Tukey’s post hoc test.

## Data Availability

The data supporting the findings of this study are presented within the manuscript.

## Abbreviations

POCD: Postoperative cognitive dysfunction
CX3CL1: C-X3-C Motif Chemokine Ligand 1
CX3CR1: CX3CL1 chemokine receptor 1
CNS: Central nervous system
GABA: Gamma-aminobutyric acid
TF: Tibial fracture
TNF-α: Tumor necrosis factor-alpha
IL-1β: Interleukin (IL)-1-beta
IL-6: Interleukin (IL)-6
CBV: Cerebral blood volume
OIS: Optical intrinsic signal
Mao B: Monoamine oxidase B
GFAP: Glial fibrillary acidic protein
Iba-1: Ionized calcium binding adapter molecule 1

## References

[1] Mashour GA, Woodrum DT, Avidan MS (2015). Neurological complications of surgery and anaesthesia. Br J Anaesth.

[2] Zenilman ME (2017). Delirium: an important postoperative complication. JAMA.

[3] Inouye SK, Marcantonio ER, Kosar CM, Tommet D, Schmitt EM, Travison TG, Saczynski JS, Ngo LH, Alsop DC, Jones RN (2016). The short-term and long-term relationship between delirium and cognitive trajectory in older surgical patients. Alzheimers Dement.

[4] Ramaiah R, Lam AM (2009). Postoperative cognitive dysfunction in the elderly. Anesthesiol Clin.

[5] Krenk L, Rasmussen LS, Kehlet H (2010). New insights into the pathophysiology of postoperative cognitive dysfunction. Acta Anaesthesiol Scand.

[6] Hovens IB, Schoemaker RG, van der Zee EA, Absalom AR, Heineman E, van Leeuwen BL (2014). Postoperative cognitive dysfunction: involvement of neuroinflammation and neuronal functioning. Brain Behav Immun.

[7] Terrando N, Eriksson LI, Ryu JK (2011). Resolving postoperative neuroinflammation and cognitive decline. Ann Neurol.

[8] Cibelli M, Fidalgo AR, Terrando N, Ma D, Monaco C, Feldmann M, Takata M, Lever IJ, Nanchahal J, Fanselow MS, Maze M (2010). Role of interleukin-1beta in postoperative cognitive dysfunction. Ann Neurol.

[9] Lai IK, Valdearcos M, Morioka K, Saxena S, Feng X, Li R, Uchida Y, Lijun A, Li W, Pan J, Koliwad S, Marcucio R, Wulff H, Maze M (2020). Blocking Kv1.3 potassium channels prevents postoperative neuroinflammation and cognitive decline without impairing wound healing in mice. Br J Anaesth.

[10] Feng X, Valdearcos M, Uchida Y, Lutrin D, Maze M, Koliwad SK (2017). Microglia mediate postoperative hippocampal inflammation and cognitive decline in mice. JCI Insight.

[11] Li Z, Cao X, Ma H, Cui Y, Li X, Wang N, Zhou Y (2018). Surgical trauma exacerbates cognitive deficits and neuroinflammation in aged rats: the role of CX3CL1-CX3CR1 signaling. J Neuropathol Exp Neurol.

[12] Turner MD, Nedjai B, Hurst T, Pennington DJ (1843). Cytokines and chemokines: at the crossroads of cell signalling and inflammatory disease. Biochim Biophys Acta.

[13] Shachar I, Karin N (2013). The dual roles of inflammatory cytokines and chemokines in the regulation of autoimmune diseases and their clinical implications. J Leukoc Biol.

[14] Old EA, Clark AK, Malcangio M (2015). The role of glia in the spinal cord in neuropathic and inflammatory pain. Handb Exp Pharmacol.

[15] Pan Y, Lloyd C, Zhou H, Dolich S, Deeds J, Gonzalo JA, Vath J, Gosselin M, Ma J, Dussault B, Woolf E, Alperin G, Culpepper J, Gutierrez-Ramos JC, Gearing D (1997). Neurotactin, a membrane-anchored chemokine u4pregulated in brain inflammation. Nature.

[16] Bazan JF, Bacon KB, Hardiman G, Wang W, Soo K, Rossi D, Greaves DR, Zlotnik A, Schall TJ (1997). A new class of membrane-bound chemokine with a CX3C motif. Nature.

[17] Landsman L, Bar-On L, Zernecke A, Kim KW, Krauthgamer R, Shagdarsuren E, Lira SA, Weissman IL, Weber C, Jung S (2009). CX3CR1 is required for monocyte homeostasis and atherogenesis by promoting cell survival. Blood.

[18] Ishida Y, Gao JL, Murphy PM (2008). Chemokine receptor CX3CR1 mediates skin wound healing by promoting macrophage and fibroblast accumulation and function. J Immunol.

[19] Auffray C, Fogg D, Garfa M, Elain G, Join-Lambert O, Kayal S, Sarnacki S, Cumano A, Lauvau G, Geissmann F (2007). Monitoring of blood vessels and tissues by a population of monocytes with patrolling behavior. Science.

[20] Clark AK, Malcangio M (2014). Fractalkine/CX3CR1 signaling during neuropathic pain. Front Cell Neurosci.

[21] Ahn JH, Kim DW, Park JH, Lee TK, Lee HA, Won MH, Lee CH (2019). Expression changes of CX3CL1 and CX3CR1 proteins in the hippocampal CA1 field of the gerbil following transient global cerebral ischemia. Int J Mol Med.

[22] Terrando N, Monaco C, Ma D, Foxwell BM, Feldmann M, Maze M (2010). Tumor necrosis factor-alpha triggers a cytokine cascade yielding postoperative cognitive decline. Proc Natl Acad Sci U S A.

[23] Skvarc DR, Berk M, Byrne LK, Dean OM, Dodd S, Lewis M, Marriott A, Moore EM, Morris G, Page RS, Gray L (2018). Post-operative cognitive dysfunction: an exploration of the inflammatory hypothesis and novel therapies. Neurosci Biobehav Rev.

[24] Zhang MD, Barde S, Yang T, Lei B, Eriksson LI, Mathew JP, Andreska T, Akassoglou K, Harkany T, Hökfelt TGM, Terrando N (2016). Orthopedic surgery modulates neuropeptides and BDNF expression at the spinal and hippocampal levels. Proc Natl Acad Sci U S A.

[25] Bolton MM, Pittman AJ, Lo DC (2000). Brain-derived neurotrophic factor differentially regulates excitatory and inhibitory synaptic transmission in hippocampal cultures. J Neurosci.

[26] Baldelli P, Hernandez-Guijo JM, Carabelli V, Carbone E (2005). Brain-derived neurotrophic factor enhances GABA release probability and nonuniform distribution of N- and P/Q-type channels on release sites of hippocampal inhibitory synapses. J Neurosci.

[27] Michels L, Martin E, Klaver P, Edden R, Zelaya F, Lythgoe DJ, Lüchinger R, Brandeis D, O’Gorman RL (2012). Frontal GABA levels change during working memory. PLoS One.

[28] Jo S, Yarishkin O, Hwang YJ, Chun YE, Park M, Woo DH, Bae JY, Kim T, Lee J, Chun H, Park HJ, Lee DY, Hong J, Kim HY, Oh SJ, Park SJ, Lee H, Yoon BE, Kim YS, Jeong Y, Shim I, Bae YC, Cho J, Kowall NW, Ryu H, Hwang E, Kim D, Lee CJ (2014). GABA from reactive astrocytes impairs memory in mouse models of Alzheimer's disease. Nat Med.

[29] Volk DW, Lewis DA (2005). GABA targets for the treatment of cognitive dysfunction in schizophrenia. Curr Neuropharmacol.

[30] Czuczwar SJ, Patsalos PN (2001). The new generation of GABA enhancers. Potential in the treatment of epilepsy. CNS Drugs.

[31] Harry LE, Sandison A, Paleolog EM, Hansen U, Pearse MF, Nanchahal J (2008). Comparison of the healing of open tibial fractures covered with either muscle or fasciocutaneous tissue in a murine model. J Orthop Res.

[32] Mills JH, Alabanza LM, Mahamed DA, Bynoe MS (2012). Extracellular adenosine signaling induces CX3CL1 expression in the brain to promote experimental autoimmune encephalomyelitis. J Neuroinflammation.

[33] Itoh J, Nabeshima T, Kameyama T (1990). Utility of an elevated plus-maze for the evaluation of memory in mice: effects of nootropics, scopolamine and electroconvulsive shock. Psychopharmacology..

[34] Sharma AC, Kulkarni SK (1992). Evaluation of learning and memory mechanisms employing elevated plus-maze in rats and mice. Progress Neuro-Psychopharmacol Biol Psychiatry.

[35] Ukai M, Miura M, Kameyama T (1995). Effects of galanin on passive avoidance response, elevated plus-maze learning, and spontaneous alternation performance in mice. Brain Research Bulletin..

[36] Miyazaki S, Imaizumi M, Machida H (1995). The effects of anxiolytics and anxiogenics on evaluation of learning and memory in an elevated plus-maze test in mice. Methods Find Exp Clin Pharmacol.

[37] Chaplan SR, Bach FW, Pogrel JW, Chung JM, Yaksh TL (1994). Quantitative assessment of tactile allodynia in the rat paw. J. Neurosci. Methods..

[38] Lee S, Kang BM, Kim JH, Min J, Kim HS, Ryu H, Park H, Bae S, Oh D, Choi M, Suh M (2018). Real-time in vivo two-photon imaging study reveals decreased cerebro-vascular volume and increased blood-brain barrier permeability in chronically stressed mice. Sci Rep.

[39] Attwell D, Buchan AM, Charpak S, Lauritzen M, Macvicar BA, Newman EA (2010). Glial and neuronal control of brain blood flow. Nature.

[40] Iadecola C (2004). Neurovascular regulation in the normal brain and in Alzheimer’s disease. Nat Rev Neurosci.

[41] Bell RD, Winkler EA, Sagare AP, Singh I, LaRue B, Deane R, Zlokovic BV (2010). Pericytes control key neurovascular functions and neuronal phenotype in the adult brain and during brain aging. Neuron.

[42] Han K, Min J, Lee M, Kang BM, Park T, Hahn J, Yei J, Lee J, Woo J, Lee CJ, Kim SG, Suh M (2019). Neurovascular coupling under chronic stress is modified by altered GABAergic interneuron activity. J Neurosci.

[43] Schmidt-Wilcke T, Fuchs E, Funke K, Vlachos A, Müller-Dahlhaus F, Puts NAJ, Harris RE, Edden RAE (2018). GABA-from Inhibition to cognition: emerging concepts. Neuroscientist.

[44] Degos V, Vacas S, Han Z, van Rooijen N, Gressens P, Su H, Young WL, Maze M (2013). Depletion of bone marrow-derived macrophages perturbs the innate immune response to surgery and reduces postoperative memory dysfunction. Anesthesiology.

[45] Vacas S, Degos V, Feng X, Maze M (2013). The neuroinflammatory response of postoperative cognitive decline. Br Med Bull.

[46] Moatti D, Faure S, Fumeron F, Amara Mel-W, Seknadji P, McDermott D, Debré P, Aumont MC, Murphy PM, de Prost D, Combadière C (2001). Polymorphism in the fractalkine receptor CX3CR1 as a genetic risk factor for coronary artery disease. Blood.

[47] Tacke F, Alvarez D, Kaplan TJ, Jakubzick C, Spanbroek R, Llodra J, Garin A, Liu J, Mack M, van Rooijen N, Lira SA, Habenicht AJ, Randolph GJ (2007). Monocyte subsets differentially employ CCR2, CCR5, and CX3CR1 to accumulate within atherosclerotic plaques. J Clin Invest.

[48] Sheridan GK, Murphy KJ (2013). Neuron-glia crosstalk in health and disease: fractalkine and CX3CR1 take centre stage. Open Biol..

[49] Ni H, Wang Y, An K, Liu Q, Xu L, Zhu C, Deng H, He Q, Wang T, Xu M, Zheng Y, Huang B, Fang J, Yao M (2019). Crosstalk between NFkappaB-dependent astrocytic CXCL1 and neuron CXCR2 plays a role in descending pain facilitation. J Neuroinflammation..

[50] Jha MK, Jo M, Kim JH, Suk K (2019). Microglia-astrocyte crosstalk: an intimate molecular conversation. Neuroscientist..

[51] Hagino S, Iseki K, Mori T, Zhang Y, Hikake T, Yokoya S, Takeuchi M, Hasimoto H, Kikuchi S, Wanaka A (2003). Slit and glypican-1 mRNAs are coexpressed in the reactive astrocytes of the injured adult brain. Glia..

[52] Santello M, Toni N, Volterra A (2019). Astrocyte function from information processing to cognition and cognitive impairment. Nat Neurosci.

[53] Ji RR, Chamessian A, Zhang YQ (2016). Pain regulation by non-neuronal cells and inflammation. Science..

[54] Gao YJ, Ji RR (2010). Targeting astrocyte signaling for chronic pain. Neurotherapeutics.

[55] Eto K, Kim SK, Takeda I, Nabekura J (2018). The roles of cortical astrocytes in chronic pain and other brain pathologies. Neurosci Res..

[56] Kim SK, Hayashi H, Ishikawa T, Shibata K, Shigetomi E, Shinozaki Y, Inada H, Roh SE, Kim SJ, Lee G, Bae H, Moorhouse AJ, Mikoshiba K, Fukazawa Y, Koizumi S, Nabekura J (2016). Cortical astrocytes rewire somatosensory cortical circuits for peripheral neuropathic pain. J Clin Invest..

[57] Eto K, Wake H, Watanabe M, Ishibashi H, Noda M, Yanagawa Y, Nabekura J (2011). Inter-regional contribution of enhanced activity of the primary somatosensory cortex to the anterior cingulate cortex accelerates chronic pain behavior. J Neurosci..

[58] Ben Haim L, Carrillo-de Sauvage MA, Ceyzeriat K, Escartin C (2015). Elusive roles for reactive astrocytes in neurodegenerative diseases. Front Cell Neurosci.

[59] Wojdasiewicz P, Turczyn P, Dobies-Krzesniak B, Frasunska J, Tarnacka B (2019). Role of CX3CL1/CX3CR1 signaling axis activity in osteoporosis. Mediators Inflamm.

[60] Donnelly DJ, Longbrake EE, Shawler TM, Kigerl KA, Lai W, Tovar CA, Ransohoff RM, Popovich PG (2011). Deficient CX3CR1 signaling promotes recovery after mouse spinal cord injury by limiting the recruitment and activation of Ly6Clo/iNOS+ macrophages. J Neurosci.

[61] McMillin M, Grant S, Frampton G, Andry S, Brown A, DeMorrow S (2016). Fractalkine suppression during hepatic encephalopathy promotes neuroinflammation in mice. J Neuroinflammation.

[62] Zhang M, Xu G, Liu W, Ni Y, Zhou W (2012). Role of fractalkine/CX3CR1 interaction in light-induced photoreceptor degeneration through regulating retinal microglial activation and migration. PLoS One.

[63] Liu Z, Chen S, Qiu C, Sun Y, Li W, Jiang J, Zhang JM (2018). Fractalkine/CX3CR1 contributes to endometriosis-induced neuropathic pain and mechanical hypersensitivity in rats. Front Cell Neurosci.

[64] Sumner P, Edden RA, Bompas A, Evans CJ, Singh KD (2010). More GABA, less distraction: a neurochemical predictor of motor decision speed. Nat Neurosci.

[65] Edden RA, Crocetti D, Zhu H, Gilbert DL, Mostofsky SH (2012). Reduced GABA concentration in attention-deficit/hyperactivity disorder. Arch Gen Psychiatry.

[66] Freria CM, Hall JC, Wei P, Guan Z, McTigue DM, Popovich PG (2017). Deletion of the fractalkine receptor, CX3CR1, improves endogenous repair, axon sprouting, and synaptogenesis after spinal cord injury in mice. J Neurosci.

[67] Girouard H, Iadecola C (2006). Neurovascular coupling in the normal brain and in hypertension, stroke, and Alzheimer disease. J Appl Physiol (1985).

[68] Lecrux C, Hamel E (2011). The neurovascular unit in brain function and disease. Acta Physiol (Oxf).

[69] Lecrux C, Bourourou M, Hamel E (2019). How reliable is cerebral blood flow to map changes in neuronal activity?. Autonomic Neuroscience-Basic & Clinical.

[70] Fergus A, Lee KS (1997). GABAergic regulation of cerebral microvascular tone in the rat. J Cereb Blood Flow Metab.

[71] Donahue MJ, Rane S, Hussey E, Mason E, Pradhan S, Waddell KW, Ally BA (2014). gamma-Aminobutyric acid (GABA) concentration inversely correlates with basal perfusion in human occipital lobe. J Cereb Blood Flow Metab.

[72] Lindia JA, McGowan E, Jochnowitz N, Abbadie C (2005). Induction of CX3CL1 expression in astrocytes and CX3CR1 in microglia in the spinal cord of a rat model of neuropathic pain. J Pain.

[73] Staniland AA, Clark AK, Wodarski R, Sasso O, Maione F, D'Acquisto F, Malcangio M (2010). Reduced inflammatory and neuropathic pain and decreased spinal microglial response in fractalkine receptor (CX3CR1) knockout mice. J Neurochem.

[74] Clark AK, Yip PK, Grist J, Gentry C, Staniland AA, Marchand F, Dehvari M, Wotherspoon G, Winter J, Ullah J, Bevan S, Malcangio M (2007). Inhibition of spinal microglial cathepsin S for the reversal of neuropathic pain. Proc Natl Acad Sci U S A.

[75] Zhuang ZY, Kawasaki Y, Tan PH, Wen YR, Huang J, Ji RR (2007). Role of the CX3CR1/p38 MAPK pathway in spinal microglia for the development of neuropathic pain following nerve injury-induced cleavage of fractalkine. Brain Behav Immun.

[76] Yin Q, Cheng W, Cheng MY, Fan SZ, Shen W (2010). Intrathecal injection of anti-CX3CR1 neutralizing antibody delayed and attenuated pain facilitation in rat tibial bone cancer pain model. Behav Pharmacol.

[77] Hu JH, Yang JP, Liu L, Li CF, Wang LN, Ji FH, Cheng H (2012). Involvement of CX3CR1 in bone cancer pain through the activation of microglia p38 MAPK pathway in the spinal cord. Brain Res.

[78] Arima Y, Kamimura D, Atsumi T, Harada M, Kawamoto T, Nishikawa N, et al. A pain-mediated neural signal induces relapse in murine autoimmune encephalomyelitis, a multiple sclerosis model. Elife. 2015;4. 10.7554/eLife.08733. 10.7554/eLife.08733 PMC4530187 26193120

[79] White GE, Tan TC, John AE, Whatling C, McPheat WL, Greaves DR (2010). Fractalkine has anti-apoptotic and proliferative effects on human vascular smooth muscle cells via epidermal growth factor receptor signalling. Cardiovasc Res.

[80] Karlstrom S, Nordvall G, Sohn D (2013). Substituted 7-amino-5-thio-thiazolo[4,5-d]pyrimidines as potent and selective antagonists of the fractalkine receptor (CX3CR1). J Med Chem.

