# Supplementary material for: Orthopedic surgery-induced cognitive dysfunction is mediated by CX3CL1/R1 signaling
Source: J Neuroinflammation. 2021 Apr 15;18:93. doi: 10.1186/s12974-021-02150-x (PMC8048361; doi:10.1186/s12974-021-02150-x)
Supplement: Supplementary file 1 — Additional file 1: Figure S1. The expression of Iba-1 in hippocampal tissue. [file 12974_2021_2150_MOESM1_ESM.docx]

**Supplementary data**

Supplementary Figure 1


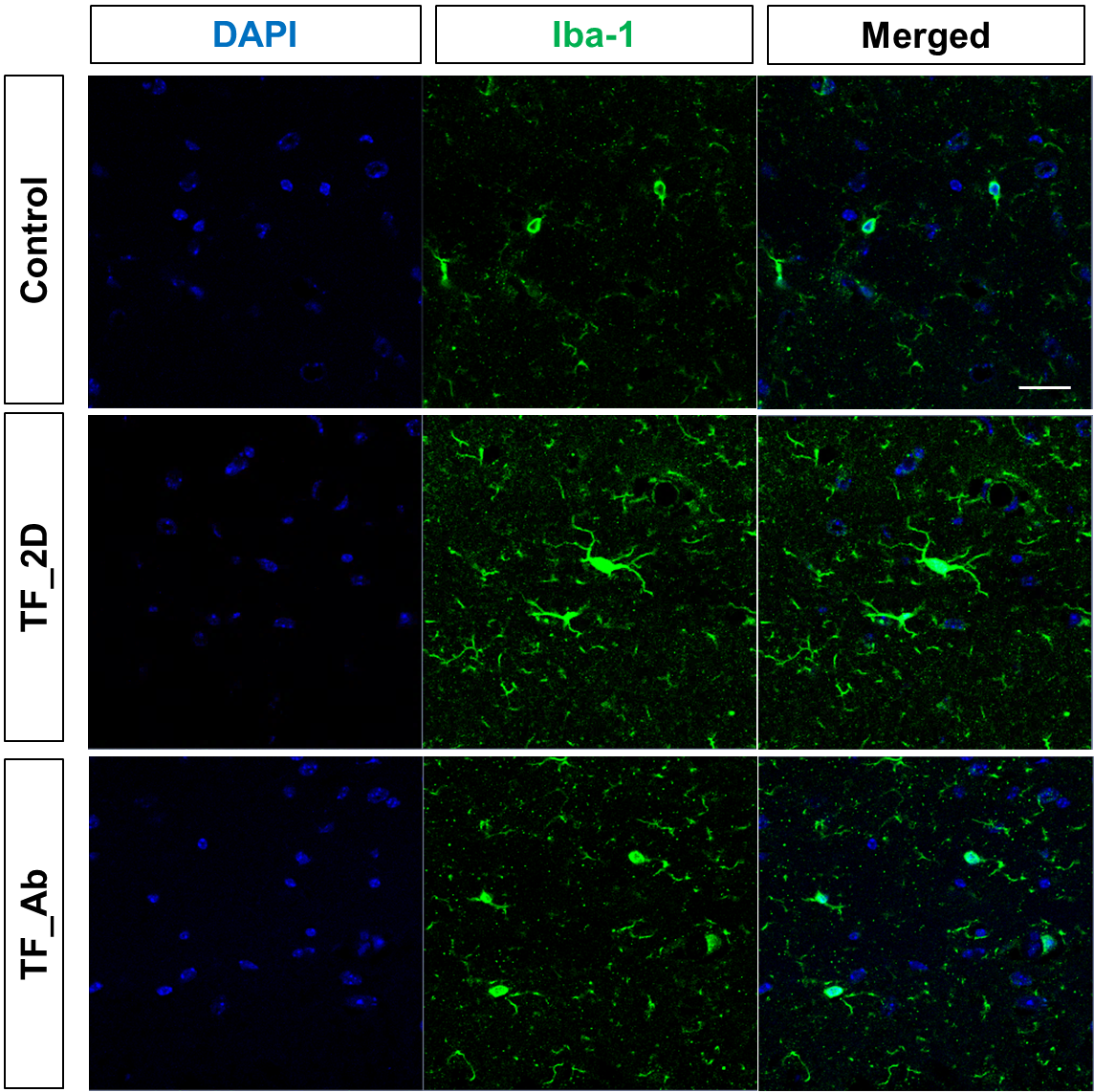


Figure S1. The expression of Iba-1 in hippocampal tissue

Immunofluorescence staining with Iba-1 B in hippocampal tissue control and sham control injected with neutralizing antibody. Microglia activation was detected in TF-induced POCD group compared to the control group. After neutralizing Ab injection, microglia activation was relived compared to the TF- induced POCD mice. TF_2D, 2 days after tibial fracture surgery group; TF_Ab, 2 days after tibial fracture surgery group injected with neutralizing Ab. Scale bar = 20 µm.
