# Supplementary material for: Orthopedic surgery-induced cognitive dysfunction is mediated by CX3CL1/R1 signaling
Source: J Neuroinflammation. 2021 Apr 15;18:93. doi: 10.1186/s12974-021-02150-x (PMC8048361; doi:10.1186/s12974-021-02150-x)
Supplement: Supplementary file 2 — Additional file 2: Figure S2. Data in Figure 4, analyzed by two-way ANOVA followed by Tukey’s post hoc test. Figure S3. Data in Figure 5, analyzed by two-way ANOVA followed by Tukey’s post hoc test. Figure S4. Data in Figure 7, analyzed by two-way ANOVA followed by Tukey’s post hoc test. [file 12974_2021_2150_MOESM2_ESM.docx]

**Supplementary data**

Supplementary Figure 2


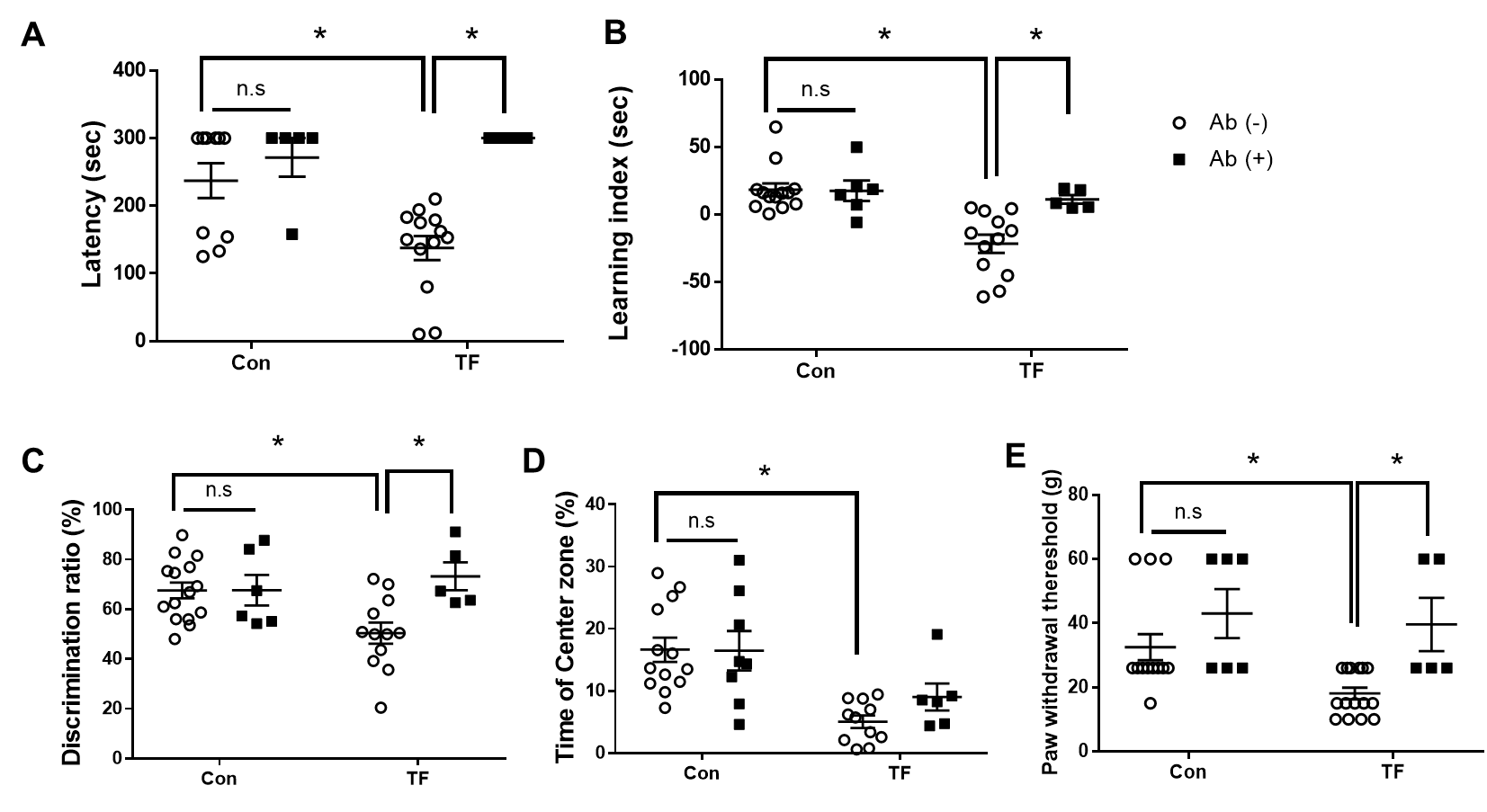


Figure S2. Data in Figure 4, analyzed by two-way ANOVA followed by Tukey’s *post hoc* test.

Cognitive dysfunction was measured using neurobehavioral tests. (A) In the passive avoidance test, the latency was significantly increased in the Ab-injected group compared to the TF-induced POCD group (p=0.0004, n=5; F (1,29)=6.519, p= 0.0162 [interaction between surgery and Ab]). (B) The learning index in the elevated plus maze test was also significantly increased in the Ab-injected group compared to the TF-induced POCD group (p=0.0153, n=5; F (1,32)=6.015, p= 0.0198 [interaction between surgery and Ab]). (C) The exploration rate for new objects in the novel objective recognition test was significantly increased in the Ab-injected group (p=0.0191, n=5; F (1,34)=5.453, p= 0.0256 [interaction between surgery and Ab]). (D) The rate of time spent in the center zone in OFT was significantly decreased in the TF-induced POCD group compared to the control group. However, it was non-significantly increased in the Ab-injected group (p=0.7920, n=5, F (1,34)=0.9101, p= 0.3468 [interaction between surgery and Ab]). (E) Von Frey test was significantly increased in the Ab-injected group compared to the TF-induced POCD group (p=0.0265, n=5; F (1,36)=1.276, p= 0.2661 [interaction between surgery and Ab]).

Supplementary Figure 3


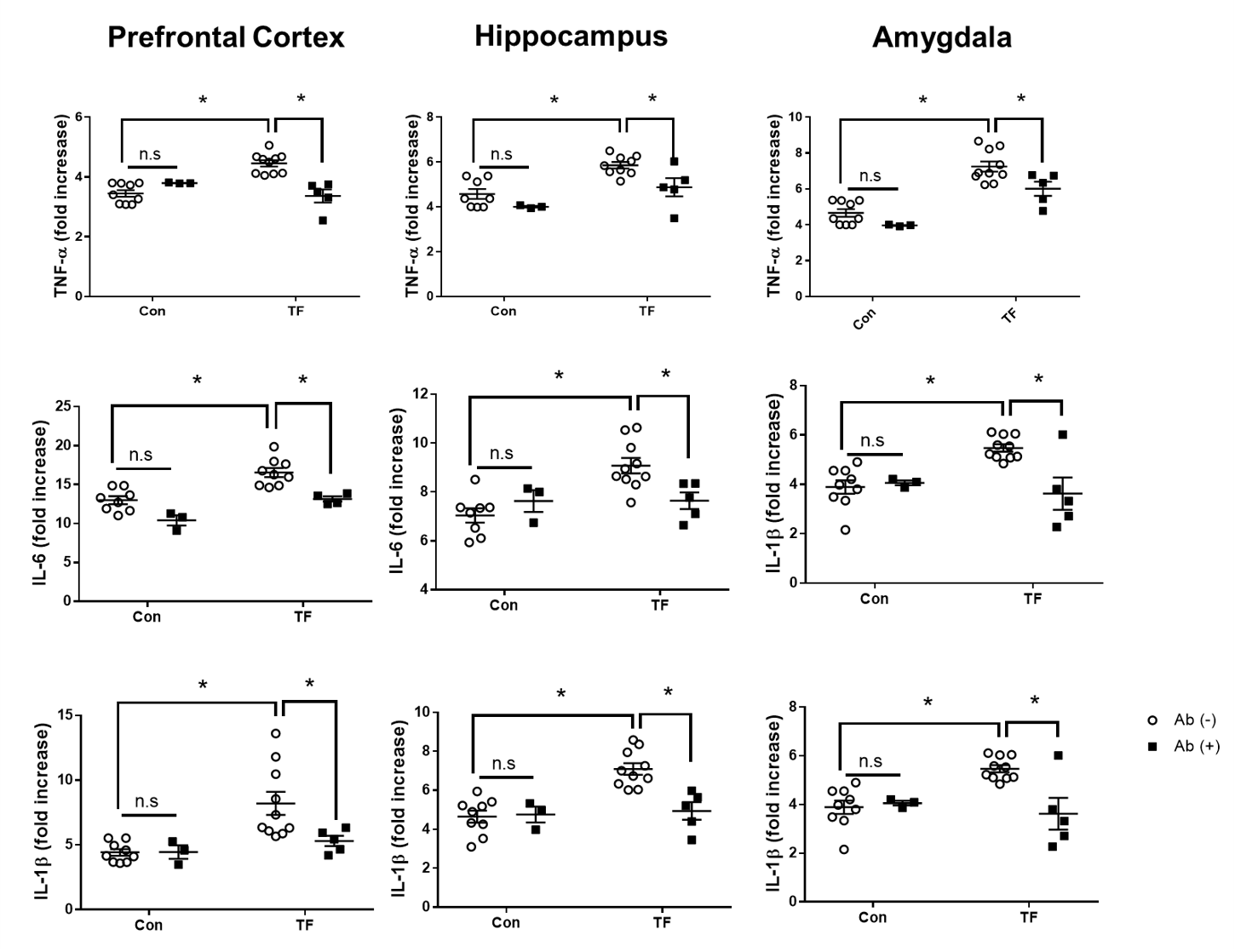


Figure S3. Data in Figure 5, analyzed by two-way ANOVA followed by Tukey’s *post hoc* test.

The inflammatory response in the brain regions were measured by ELISA analysis. Overall, decreased levels of inflammatory cytokines including TNF-α and IL-6 were detected in the brain regions of Ab-injected mice compared to the TF group. Also, IL-1β expression was significantly decreased in the prefrontal cortex, the hippocampus, and the amygdala regions in the Ab-injected group compared to the TF-induced POCD group (IL-1β in hippocampus; F (1,23)=10.73, p= 0.0033 [surgery]; F(1,23)=6.553, p=0.0175 [Ab]; F(1,23)=7.971, p=0.0096 [interaction between surgery and Ab]).

Supplementary Figure 4


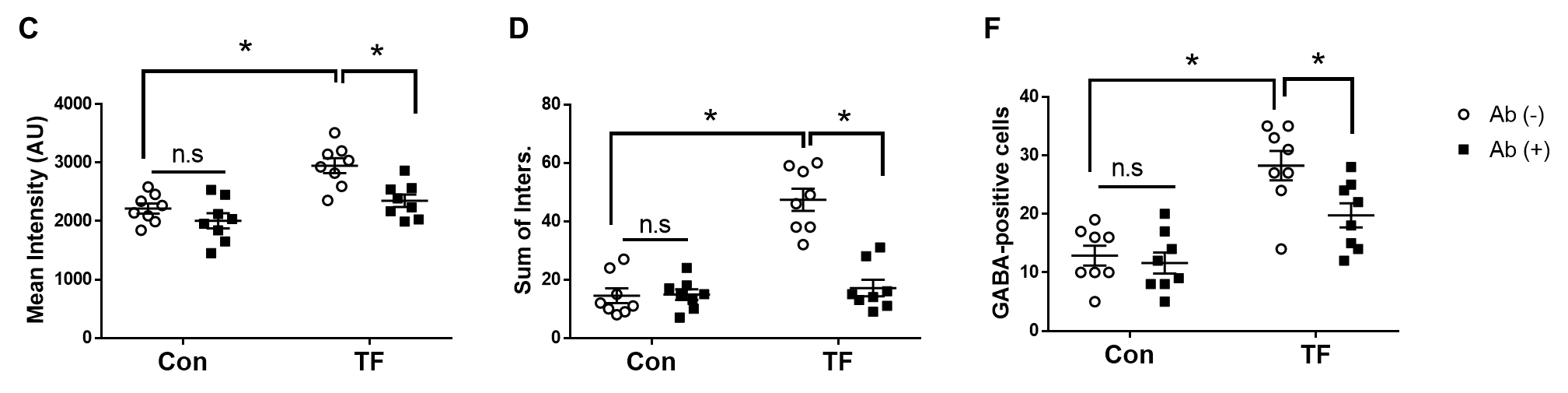


Figure S4. Data in Figure 7, analyzed by two-way ANOVA followed by Tukey’s *post hoc* test.

The alteration of astrocyte morphology and GABA expression were detected by immunohistochemistry. In TF group, increased (C) mean intensity (p=0.005, n=8), (D) intersection of astrocytes (p<0.0001, n=8), and (F) increased GABA-positive cells (p<0.0001, n=8) were detected compared to the control. In Ab-injected group, decreased (C) mean intensity (p=0.004, n=8) and (D) intersection of astrocytes (p<0.0001, n=8) and decreased of (F) GABA-positive cells (p=0.036, n=8) were detected compared to the TF group. control decreased levels of inflammatory cytokines including TNF-α and IL-6 were detected in the brain regions of mice (sum of intersection; F (1,28)=38.48, p<0.0001 [surgery]; F(1,28)=27.83), p<0.0001 [Ab]; F(1,28)=29.25, p<0.0001 [interaction between surgery and Ab]).
